# Quantitative analysis of lung lesions using unenhanced chest computed tomography images

**DOI:** 10.1111/crj.13759

**Published:** 2024-05-07

**Authors:** Fariba Zarei, Payam Jannatdoust, Siamak Malekpour, Mahshad Razaghi, Sabyasachi Chatterjee, Vani Varadhan Chatterjee, Amirbahador Abbasi, Rezvan Ravanfar Haghighi

**Affiliations:** ^1^ Medical Imaging Research Center Shiraz University of Medical Sciences Shiraz Iran; ^2^ Department of Radiology Shiraz University of Medical Sciences Shiraz Iran; ^3^ School of Medicine Tehran University of Medical Sciences Tehran Iran; ^4^ Student Research Committee Shiraz University of Medical Sciences Shiraz Iran; ^5^ Ongil (or Retired Scientist From Indian Institue of Astrophysics, Bengluru) Salem India; ^6^ Department of Instrumentation and Applied Physics Indian Institute of Science Bengaluru India

**Keywords:** chest CT, lung, pulmonary nodules, radiology

## Abstract

**Introduction:**

Chest radiograph and computed tomography (CT) scans can accidentally reveal pulmonary nodules. Malignant and benign pulmonary nodules can be difficult to distinguish without specific imaging features, such as calcification, necrosis, and contrast enhancement. However, these lesions may exhibit different image texture characteristics which cannot be assessed visually. Thus, a computer‐assisted quantitative method like histogram analysis (HA) of Hounsfield unit (HU) values can improve diagnostic accuracy, reducing the need for invasive biopsy.

**Methods:**

In this exploratory control study, nonenhanced chest CT images of 20 patients with benign (10) and cancerous (10) lesion were selected retrospectively. The appearances of benign and malignant lesions were very similar in chest CT images, and only pathology report was used to discriminate them. Free hand region of interest (ROI) was inserted inside the lesion for all slices of each lesion. Mean, minimum, maximum, and standard deviations of HU values were recorded and used to make HA.

**Results:**

HA showed that the most malignant lesions have a mean HU value between 30 and 50, a maximum HU less than 150, and a minimum HU between −30 and 20. Lesions outside these ranges were mostly benign.

**Conclusion:**

Quantitative CT analysis may differentiate malignant from benign lesions without specific malignancy patterns on unenhanced chest CT image.

## Graphical abstract

Computed tomography (CT) histogram analysis of lung lesions' Hounsfield units may differentiate malignant from benign lesions. Most malignant lesions have a mean Hounsfield unit value between 30 and 50, a maximum Hounsfield unit less than 150, and a minimum Hounsfield unit between −30 and 20. Lesions outside these ranges were mostly benign.

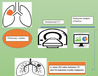

## 1 INTRODUCTION

Pulmonary neoplasms constitute a significant health problem that causes the highest number of cancer‐related mortality in both genders in many countries, including in Iran. Although several known risk factors for lung cancers can be used for risk stratification and choosing the population at most risk, unlike colorectal and breast cancers, there is no efficient method to screen for lung cancers. Most of these malignancies are discovered in moderate or late stages.^1^ However, some screening programs have been introduced that cover people with high‐risk profiles, such as a history of smoking and population that is older than 55 years. These programs are to be beneficial as they help early diagnosis.^2^, ^3^, ^4^

Diagnosis of pulmonary cancers relies primarily on computed tomography (CT) scans.^4^ Furthermore, pulmonary nodules can often be found incidentally during a chest x‐ray radiography or chest CT scan, which usually needs further investigation by high‐resolution CT imaging.^4^ The ability to differentiate malignant nodules from benign ones is a significant concern for diagnostic radiology. This is because the gold standard for the diagnosis is biopsy which is invasive and harmful and can pose risk for patients such as pneumothorax.^5^, ^6^ Further, high number of false positives is a significant challenge in this field.^7^

Although there are specific patterns of radiologic findings, such as temporal alteration in the follow‐up, calcification patterns, and some more specific findings, such as lobulation or necrosis that are pathognomic for particular types of malignant or benign lesions, a considerable number of nodules do not have any of these features. This makes it hard to rule out malignancy in many nodules if one depends merely on radiologic images.^8^, ^9^ For instance, nodules with only ground‐glass opacity appearance or ones with a subsolid component are harder to judge visually by CT scans.^10^

However, in addition to features like calcification patterns, necrotic lesions, and lesions with specific patterns of temporal alterations, some other features can also be identified by CT imaging. Some of these features are not as easily detectable by visual inspection but can guide the radiologist to a better diagnosis of the nature of the lesion. Due to the high complexity of these patterns and their undetectability in visual inspections, computer‐aided diagnosis (CAD) can be helpful in this field by objectively analyzing the shape and texture features of the nodules.^11^ For instance, CAD has been utilized to measure the heterogeneity of lesion,^12^ and it has been repeatedly reported that heterogeneity within the mass is correlated with more malignant features.^13^

Hounsfield unit (HU) is a measure of linear x‐ray attenuation within the tissue, normalized to the linear attenuation coefficient of water, which is universally utilized in mapping CT scan grayscale images. For the first time, Sieghorn et al. proposed that in the absence of calcification, HU measurement can be utilized in differentiating benign pulmonary nodules from malignant ones.^14^ Alterations of the HU after injection of contrast agents can also be measured objectively to diagnose the malignant features. For instance, it was previously reported that an enhancement of lesion (higher HU than un‐enhanced) after the injection is usually observed in malignant lesions (as a result of absorbing or entering the contrast agent into the lesion). In contrast, an enhancement of less than 15 HU is mainly seen in benign lesion.^15^ Histogram analysis (HA) allows them to objectively report the number of points in a region of interest (ROI) based on their HU numbers. It will enable us to examine the heterogeneity of these points, their mean and median amounts, and the numbers of each percentile and maximums and minimums. Furthermore, HA visualizes patterns of HU numbers which can also be used in multifactorial machine learning‐based approaches.^16^

HA of pulmonary nodules, regarding their malignant or benign nature, has been conducted in a limited number of studies before. These studies showed that features of the lesion, such as mean HU, maximum HU, or value of HU in some percentiles, correlate significantly with the histopathologic features of the lesion.^17^, ^18^, ^19^

With regard to the idea, developing an HA‐based approach to diagnosing malignant lesions, especially in cases where there are no certain signs of malignancy or benign nature, can be much more helpful in diagnosing the malignant pulmonary nodules faster and reaching better therapeutic results. This study aims to analyze the histogram of HUs in a number of malignant and benign lesions in a center in Iran to evaluate the potential role of different histogram‐driven features in diagnosing malignant tumors.

## 2 MATERIALS AND METHODS

This retrospective study used the chest CT images without contrast enhancement from Picture Archiving and Communication System (PACS). Patients were scanned by Dual slices Spirit CT (Siemens, Forchheim, Germany). The scanning protocol used 130 kVp and automatic exposure control (automatic tube current modulation). The slice thicknesses of CT images were 3 and 5 mm.

The selected data entered for this study consisted of multislice sections of 10 malignant lesions, their malignancy being confirmed by pathology study. These values were compared with the same number of cases for benign lesions. The chest CT images of the benign (such as anthracosis) and malignant (such as adenocarcinoma) lesions were not distinguishable by visual evaluation. To reach this similarity, lesions with characteristic findings for malignancy or benign nature, such as those with radiologic evidence for necrosis and certain calcification patterns, were excluded. The criteria for selection of benign cases, with similar appearance to malignant in CT images, were the pathology report. The pathology was used to prove that the lesion was benign. We have followed the patients' documents (from PACS) up to 2 years to become sure about the stability of the lesion's size.

The HU values of the lung lesions were measured by selecting the free hand ROI, without any contamination with neighboring structures. In this retrospective study, the CT images of 20 patients with benign and cancerous lung lesion, 10 for each group, were selected on the basis of pathology report. The selected ROI contained most parts of the lesion. HU values of all the slices inside each lesion were used for the analysis. Minimum, maximum, mean and standard deviations of each ROI (HU values) were recorded for HA by MATLAB program.

## 3 RESULTS

**FIGURE 1:**
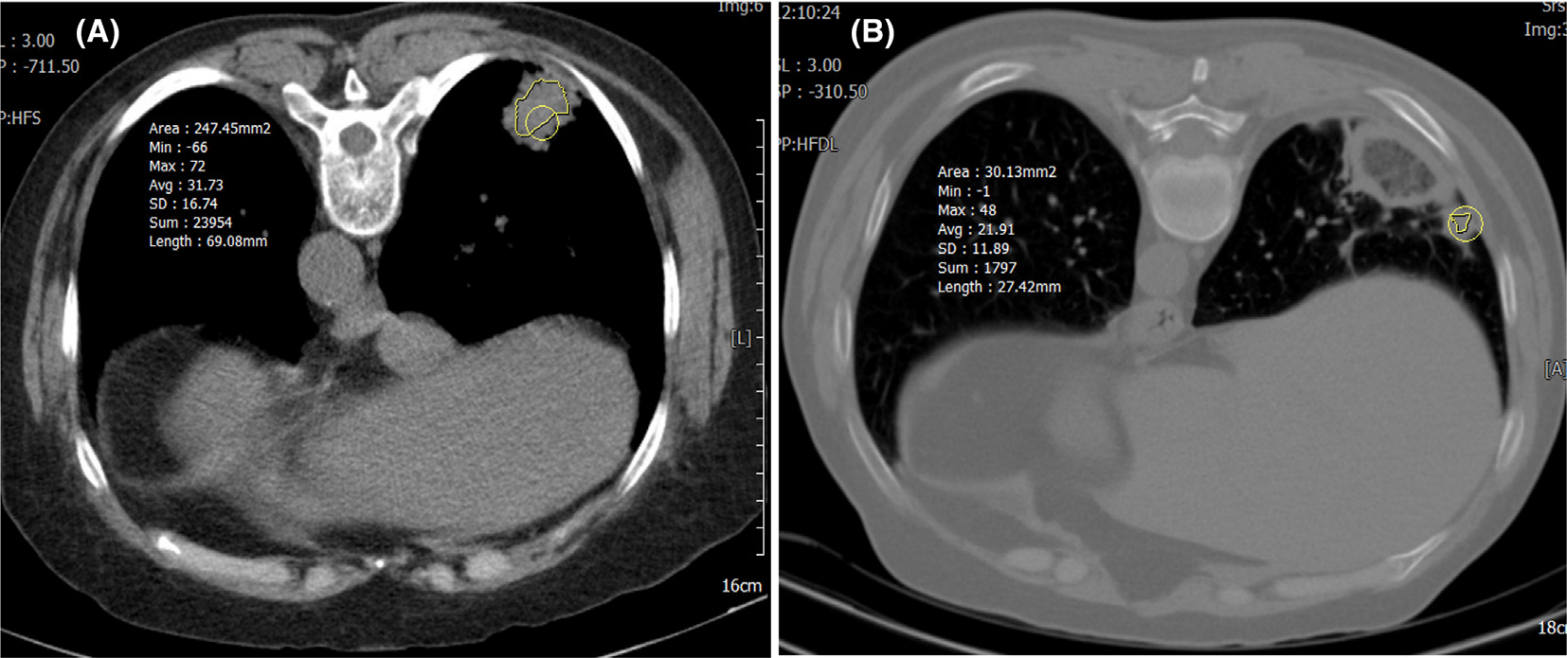
Unenhanced axial computed tomography (CT) image of (A) 72‐year‐old female having squamous cell carcinoma, (B) 41‐year‐old male having benign lesion, focal interstitial chronic inflammation.

**FIGURE 2:**
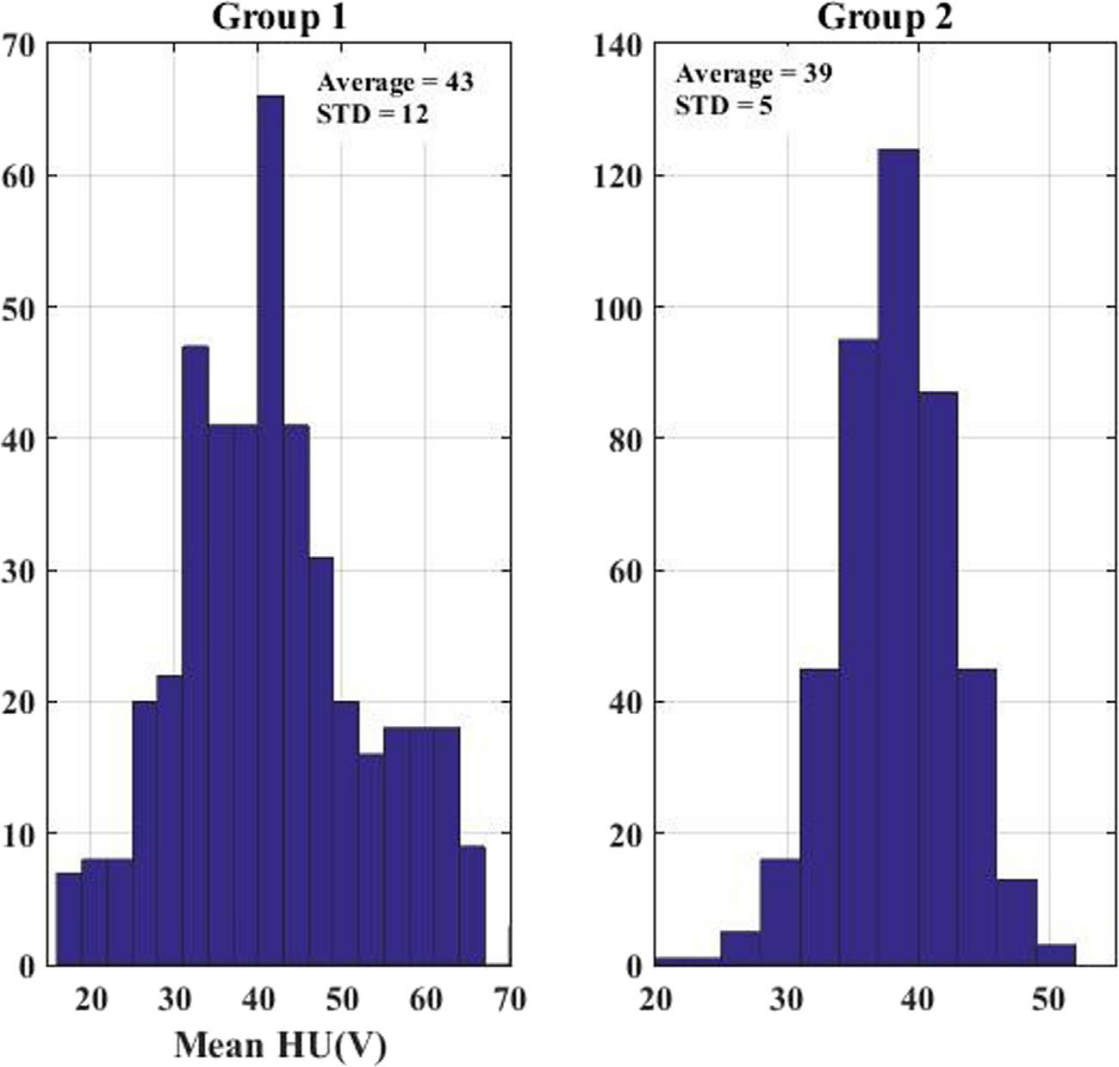
Histogram chart of mean of Hounsfield unit (HU) (V) values of group 1 (benign lesion) and group 2 (malignant lesion) for 10 patients each.

**FIGURE 3:**
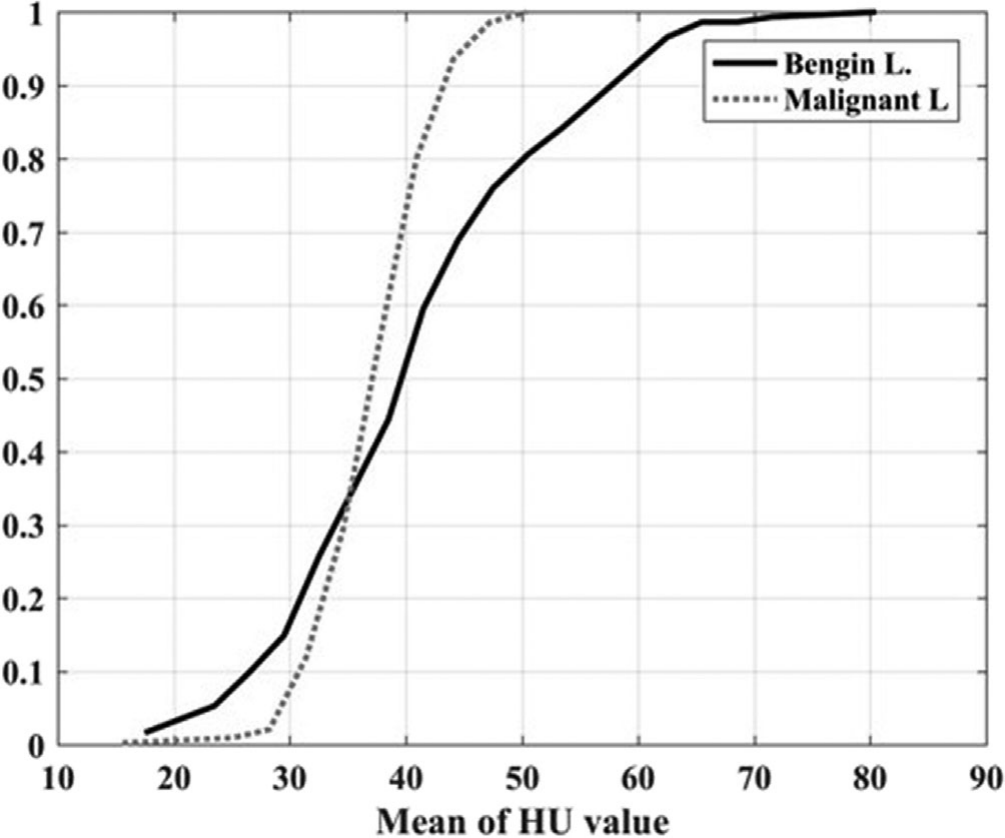
Normalized cumulative distribution of mean Hounsfield unit (HU) values of benign (group 1) and malignant (group 2) lesions.

HU values of 430 slices from group 1 (belonging to benign lesion) and group 2 (belonging to malignant lesion) were extracted from chest CT images of 20 patients (10 for each group). It can be seen a sample of malignant and benign lesions, in axial CT images of the lung, in Figure 1A,B. Histogram and cumulative distribution of HU mean, maximum, minimum and standard deviation were recorded. The histogram and cumulative distribution of the mean HU values of benign and cancerous lesions are shown in Figures 2A,B and 3A,B, respectively, as samples. It has to be noted that the *x*‐axis in these figs represent HU values, while *y*‐axis shows the frequency distribution in Figure 2A,B and normalized frequency distribution in Figure 3A,B.

Figures 2 and 3 show that the differences between the mean HU values of group 1(benign lesions) and 2 (malignant lesions) are significant. Cumulative distributions analysis in Figure 3 shows that the mean HU values of benign (group 1) and cancer (group 2) lesions cross each other, that is, at the point where the cumulative probability of occurrence of both of these lesions are about 35%. The differences between cumulative distribution curves of the mean HU values increase considerably for HU values greater than 35. The probability of the tumor (group 2) reaches close to 100% for the mean HU values at about 48, while the mean HU value of benign lesion (group 1) occurs at 70. The results of histogram and cumulative distribution analysis showed that the mean and maximum HU values may help to differentiate the type of lesions (benign from malignant lesions), while the HU(V) distributions between group 1 and 2 are not considerably different for minimum HU values. Also, the standard deviation of the HU values is slightly higher in benign lesion (group 1), implying that this type of lesion is more heterogeneous than those of cancerous ones.

We define the following:

*N*_*p*_ = number times that a lesion of type 1 (benign lesion) has a HU value within the given HU bin.

*N*_*q*_ = number of times that a lesion of type 2 (malignant lesion) has a HU value within the given HU bin.

$$N=N_{p}+N_{q}.$$

p=Np/N.

q=Nq/N.

Clearlyp+q=1.

With the following condition:

*p =* probability density of lesion 1 being in that particular HU bin.

*q =* Probability density of lesion 2 being in that particular HU bin.

We further define the percentage probabilities,

$$\%p=\left[N_{p}/N\right]\times 100\%$$

$$q=\left[N_{q}/N\right]\times 100.$$

From our observed data, we plot the *p* and *q* dependences on the HU values.

**FIGURE 4:**
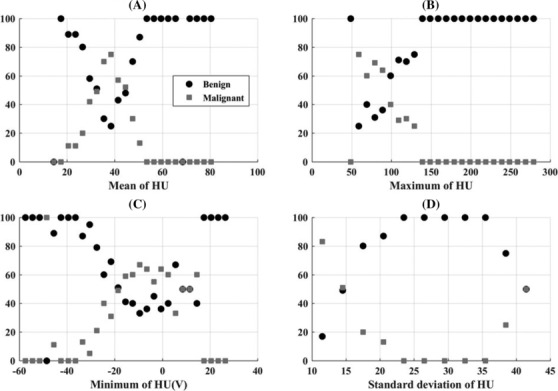
Scatter diagram of probability distribution of (A) mean, (B) maximum, (C) minimum, (D) standard deviation of Hounsfield unit (HU) value of benign (group 1) and malignant (group 2) lesions.

The results of probability distributions for mean, maximum, minimum and standard deviation of HU values of group 1 (benign lesion) and group 2 (malignant lesion) are shown by scatter diagrams in Figure 4A–D.

The probability distributions of mean, minimum and standard deviation of HU value are not monotonic. In these probabilities, we see that they can go up and come down (or vice versa) on increasing HU. This particular observation has to be understood on the basis of the chemical compositions of these lesions. Unfortunately, the compositions are not independently known. We give a qualitative description of the results.

The results of the scatter diagram of mean HU values (Figure 4A) showed that for group1 or benign lesion, *p* is greater than 90% and is dominant in the range (1) less than 30 and (2) greater than 50 HU values. The mean HU values of group 2 (malignant) are clustered around 30 to 50 HU values.

The scatter diagram of maximum HU (Figure 4B) of group 1 has the probability distribution equal to 100% for HU values greater than 100. Maximum HU values have the probability distribution between 30% and 70% for the HU values between 90 and 110 in group 2.

The scatter diagram of the minimum HU values (Figure 4C) of group 1 predominantly has the probability distribution values greater than 80% for HU values less than −30. The minimum HU values of group 2 lie between −20 and 0.0 with the probability distribution greater than 40 and less than 80%. These are empirical observations of the data. To understand their basis fully, independent investigations to find the compositions of the lesions will be necessary.

The probability distribution of standard deviation of HU values (Figure 4D) for group 1 is 20% for HU value equal to 10. Then, probability distribution of standard deviation increases gradually and reaches 100% for HU values greater than 20. Scatter diagram shows that the HU values of standard deviation between 10 and 20 have the probability distributions of 80% to 20% in the lesion of group 2.

## 4 DISCUSSION

The present study was aimed to evaluate the quantitative or HA to discriminate benign from malignant lesions in unenhanced chest CT images. As described in the results, our analysis showed that the trend within the values of mean, maximum, and minimum HU of the malignant lesions differed from those of benign lesions in the quantitative analysis, HA, of the results. Our analysis shows that most malignant pulmonary nodules have a mean HU within 30 and 50, and lesions with a mean HU outside these limits are most likely benign. Moreover, our results depict that malignant lesions most likely show a maximum HU of less than 150, and nodules with a maximum HU higher than 150 in the HA could be considered as benign. These results lead us to propose that computer‐automated analysis of unenhanced CT imaging findings can be a helpful tool that can help distinguish malignant lesions from benign ones. Similarly, lesions with malignant nature usually show a minimum within ROI HU between −30 and 20, and those with minimum values out of this spectrum are more likely to be benign.

In other words, our study shows that, in case of a pulmonary nodule that cannot be identified as malignant or benign based on visual inspection, computerized HA can be a valuable tool in choosing the patients for biopsy. In this setting, nodules with mean HU less than 30 or higher than 50, maximum HU of more than 150, or minimum HU less than −30 or more than 20 can be considered benign and a biopsy need not be essential. Based on the present findings, CT quantitative analysis of pulmonary nodules may be a useful tool to discriminate benign from malignant lesions in unenhanced chest CT images. Therefore, this analysis can be developed as a computerized scoring system that helps radiologists differentiate lesions.

Our findings support previous evidence in the existing literature that has shown the role of HA in indicating malignancy. Similar to our research, the mean HU value of the lesions has been demonstrated in several previous articles as a indicator of malignancy.^17^, ^20^ Another quantitative feature with diagnostic ability in our work was the maximum CT values and similar to mean HU values; this finding also reproduced results from the previous research that demonstrated the diagnostic performance of maximum HU values and 100th percentile HU.^19^ Moreover, our study showed a lower diagnostic power for minimum HU values, which were not reported in previous studies.

Although not present in our analysis, previous literature has revealed that 75th percentile HU values can also be a good predictor of invasiveness and malignancy.^21^, ^22^ However, although standard deviation and entropy also yielded good diagnostic ability in some previous studies,^20^, ^22^ standard deviation did not show a good diagnostic ability in our work. Furthermore, peak HU value and its alterations during follow‐up could also be a good predictor of malignant transformation in one study.^23^ HA of HU was not the only computerized CT scan analysis assessed in previous literature for diagnosing malignant from benign lesions. For instance, evidence shows that nodule diameter and size can also be utilized in addition to HA results for increasing the accuracy of prediction.^17^, ^18^ Furthermore, a deep learning algorithm that comparatively used HA and computerized texture analysis results showed a better performance of heterogeneity measure of texture analysis in predicting malignancy.^16^

Also, the results of a retrospective study done by Digumarthy et al. showed that the malignant lesions can be distinguished from benign one by radiomic analysis. The results of radiomic analysis of CT images of subsolid lung lesions revealed interim variation for malignant lesions.^24^ The results of the present study showed that HA is able to predict the nature of lung lesions (benign from malignant).

Furthermore, some studies used deep learning methods to assess the ability of such algorithms in diagnosing malignant lesions based on a complex combination of several derivatives of computerized CT analysis and showed that these methods yield a good diagnostic power.^25^, ^26^, ^27^ For instance, one study showed that a deep neural network of CT scan results shows a better diagnostic performance than board‐certified radiologists in identifying adenocarcinomas among ground‐glass opacity nodules.^25^

This approaches can be improved by doing more statistical analysis as will be reported in our future publication.

## 5 CONCLUSION

HA of mean, minimum, and maximum of HU value may be used to discriminate benign and malignant lesions, with similar appearance in unenhanced chest CT images. This study shows that the lesions with mean HU value less than 30 or higher than 50 are most likely not malignant. Similarly, lesions with a maximum HU of more than 150 are mostly benign. Furthermore, most malignant lesions have a minimum HU between −30 and 20. These results can be helpful in reducing unnecessary biopsies by forming scoring systems that predict the possibility of malignancy, based on a combination of these measures.

## AUTHOR CONTRIBUTIONS

**Fariba Zarei:** Coordinator and inputs for radiological and diagnostic studies. **Payam Jannatdoust:** Data curation and writing – original draft. **Siamak Malekpour:** Data collection and experimental study. **Mahshad Razaghi:** Edited and finalized manuscript and data curation. **Sabyasachi Chatterjee:** Mathematical and theoretical works. **Vani Varadhan Chatterjee:** Statistical analysis. **Amirbahador Abbasi:** Edited and finalized manuscript and data curation. **Rezvan Ravanfar Haghighi:** Statistical analysis, results interpretation, and writing the first draft of the manuscript.

## CONFLICT OF INTEREST STATEMENT

The authors have no conflict of interest. The authors have performed this work in their academic and their professional interest. They are not supported by any agency for this work, and full credit would be given to their own institutions. All the authors from Iran are supported by the grant (no. 15559) from their university which is acknowledged. The authors from India also have no conflict of interest and are not supported by any agency for this work.

## ETHICS STATEMENT

Ethical committee code of the present study is SUMS.MED.REC.1397.428.

## PATIENT CONSENT STATEMENT

This study used chest CT images saved at Picture Archiving and Communication System (PACS), Infinite. Therefore, the consent form was not needed.

## PERMISSION TO REPRODUCE MATERIAL FROM OTHER SOURCES

We did not use material from other sources in this study.

## CLINICAL TRIAL REGISTRATION

This study was not a clinical trial.

## Data Availability

Data from this study are available.
